# Sex-Related Shape Variation and Right–Left Asymmetry in the Stylopodium and Zeugopodium of Guinea Pigs

**DOI:** 10.3390/ani15243636

**Published:** 2025-12-17

**Authors:** Edyta Pasicka, Iliana Stefanova Ruzhanova-Gospodinova, Seven Mustafa, Ana Pesic, Ebuderda Günay, Nicoleta Manuta, Barış Can Güzel, Ebru Eravci Yalin, Ozan Gündemir

**Affiliations:** 1 Department of Biostructure and Animal Physiology, Faculty of Veterinary Medicine, Wrocław University of Environmental and Life Sciences, 50-375 Wroclaw, Poland; 2 Department of Anatomy, Physiology and Animal Sciences, University of Forestry, 1797 Sofia, Bulgaria; iliana_ruzhanova@ltu.bg; 3 Department of Surgery, Radiology, Obstetrics, and Gynecology, University of Forestry, 1797 Sofia, Bulgaria; s.mustafa@ltu.bg; 4 Department of Equine, Small Animal, Poultry and Wild Animal Diseases, Faculty of Veterinary Medicine, University of Belgrade, 11000 Belgrade, Serbia; ana.pesic@vet.bg.ac.rs; 5 Department of Wild Animal Disease and Ecology, Faculty of Veterinary Medicine, Istanbul University-Cerrahpasa, Istanbul 34320, Türkiye; ebuderda.gunay@iuc.edu.tr; 6 Institute of Graduate Studies, Istanbul University-Cerrahpasa, Istanbul 34320, Türkiye; nicoletamanuta@ogr.iuc.edu.tr; 7 Department of Anatomy, Faculty of Veterinary Medicine, Siirt University, Siirt 56100, Türkiye; baris.guzel@siirt.edu.tr; 8 Department of Surgery, Faculty of Veterinary Medicine, Istanbul University-Cerrahpasa, Istanbul 34320, Türkiye; ebru.eravciyalin@iuc.edu.tr; 9 Department of Anatomy, Faculty of Veterinary Medicine, Istanbul University-Cerrahpasa, Istanbul 34320, Türkiye

**Keywords:** forelimb–hindlimb comparison, fluctuating asymmetry, geometric morphometrics, limb bones, sexual dimorphism

## Abstract

The aim of this study was to identify the presence and magnitude of right–left asymmetry in the fore- and hindlimb long bones of adult guinea pigs (*Cavia porcellus*), and, in parallel, to evaluate sex-related differences in shape and size in the same bones. For this purpose, the stylopodium and zeugopodium bones were analyzed together. Computed tomography data were obtained from all animals under a standard protocol, three-dimensional bone models were generated from these datasets, and these models formed the basis for the geometric morphometric analyses. The analyses showed that sex accounted for a small but consistent proportion of shape variation (approximately 5–7%) in the antebrachium, femur and crus, whereas this effect was more limited in the humerus. Asymmetry analysis based on paired right–left data revealed that directional asymmetry was generally low, whereas fluctuating asymmetry varied across bones. The highest asymmetry was detected in the antebrachium, the crus showed an intermediate level, and the stylopodial elements, humerus and femur, were comparatively more symmetrical. This pattern is consistent with the biomechanical expectation that small right–left deviations tend to accumulate in distal and functionally more active forelimb segments that are positioned farther from the trunk. In conclusion, the findings indicate that asymmetry arises gradually as a function of bone position and functional use rather than from a single growth error, and that distal forelimb elements should therefore be given particular attention in asymmetry studies.

## Simple Summary

Long-bone segments that are positioned farther from the trunk generally experience more complex movements and loading conditions, the range and complexity of motion also increase, and the loads transmitted by muscles and tendons to the bone surface become more variable; this suggests that asymmetry may be more pronounced in distal segments. In this study, we tested exactly this idea by using previously acquired 3D CT images of adult, clinically healthy guinea pigs. When the left side of the same bone was compared with the contralateral (right) bone using the standard protocol, we found that the forearm bone (antebrachium) showed slightly greater right–left differences than the other long bones. In contrast, bones located closer to the trunk (that is, the humerus and femur) were more symmetrical. Although there were some size differences between females and males, this was not the main cause of the asymmetry observed. These findings show that even in healthy animals, small right–left shape differences can occur, and that these differences are related to the position of the bone and its functional use.

## 1. Introduction

The domestic guinea pig (*Cavia porcellus* Linnaeus, 1758) is a hystricomorph rodent whose origin and domestication history, together with its cytogenetic background, have been well documented in the literature [1,2]. Beyond its zoological relevance, *C. porcellus* has a long-standing role as a biomedical model, having been used for decades in experimental studies of infectious and non-infectious diseases, including viral infections and tuberculosis research [3,4,5,6]. This combination of practical availability, established clinical imaging protocols, and translational value makes the guinea pig a useful species for investigating subtle aspects of musculoskeletal variation under controlled (clinically healthy) conditions.

Although bilaterally organized organisms share a fundamentally symmetrical body plan, perfect right–left identity is rarely achieved. Small, random deviations between sides are commonly described as fluctuating asymmetry (FA), whereas consistent side-biased differences are referred to as directional asymmetry (DA) [7,8,9]. In skeletal structures, asymmetry can reflect developmental instability as well as cumulative effects of functional use and mechanical loading during growth, making long bones especially informative targets for asymmetry research [7,8,9]. In this context, geometric morphometrics provides a powerful framework for quantifying shape variation in a multivariate and anatomically coherent way, enabling the separation of size-, shape-, and symmetry-related components of variation [10,11].

Bilateral asymmetry in long bones has been reported across multiple mammalian taxa. Across a wide range of organisms and biological structures, studies using geometric morphometrics suggest that fluctuating asymmetry is often detectable, whereas directional asymmetry tends to be weaker and more context-dependent, varying with the trait examined and the functional or developmental conditions under which it is expressed [12,13,14,15,16,17,18,19,20]. However, many previous investigations have assessed asymmetry trait-by-trait or bone-by-bone, and fewer studies have explicitly contrasted asymmetry levels across limb segments (e.g., stylopodium vs. zeugopodium) within a single species using fully 3D shape data [12,13,14,15,16,17]. From a biomechanical perspective, distal segments may be expected to show greater asymmetry because they are subjected to a wider range of movements and potentially more heterogeneous loading regimes than proximal elements [7,8,9].

Accordingly, this study aimed to quantify right–left asymmetry (FA and DA) in the stylopodium and zeugopodium long bones of adult, clinically healthy guinea pigs. Specifically, we analyzed the humerus and femur (stylopodium) together with the antebrachium (radius–ulna segment) and crus (tibia–fibula segment). We hypothesized that asymmetry would be more pronounced in distal elements relative to more proximal long bones. In addition to asymmetry, we also evaluated sex-related differences in bone shape and overall size (centroid size) to determine whether sexually dimorphic patterns overlapped with, or were independent from, lateralized variation.

## 2. Materials and Methods

### 2.1. Samples

The material consisted of retrospectively collected CT images from clinically normal adult guinea pigs whose scans had previously been acquired for routine diagnostic purposes. No additional imaging or handling was performed specifically for this study, and written authorization to use these existing images for research was obtained from the institution responsible for the animals. At the time of the original examinations, all individuals underwent a complete clinical evaluation by veterinarians, and only those without evidence of systemic disease, locomotor impairment, previous trauma, or skeletal deformity were imaged and thus became available for the present analysis. To obtain three-dimensional data suitable for landmark-based morphometric assessment of the appendicular skeleton, the thoracic and pelvic limbs were scanned with a 128-slice CT system (Siemens Definition AS Plus; Siemens Healthineers, Forchheim, Germany) under a single, standardized acquisition protocol. Scans were acquired with 0.6 mm slice thickness, 120 kVp tube voltage, and 240 mAs tube current, and these parameters were kept constant across animals to ensure comparability of linear and shape measurements among bones. During image acquisition, animals were positioned in sternal recumbency with forelimbs and hindlimbs extended and aligned to the median plane. All CT datasets were later reviewed by board-certified veterinary radiologists, and images showing congenital anomalies, fractures, proliferative or lytic bone lesions, or any other condition that could affect long-bone morphology were excluded. Only datasets from healthy individuals were retained for 3D landmarking of the humerus, antebrachium (radius–ulna segment), femur, and crus (tibia–fibula segment).

A total of 30 adult, clinically healthy guinea pigs were initially available for evaluation; however, not all CT volumes could be used for every bone after 3D reconstruction and automatic landmarking. Specimens in which reconstruction artefacts were observed, in which the automatic procedure captured non-anatomical CT residues as landmarks, or in which the left element could not be matched reliably with the contralateral (right) element after standardization of orientation were excluded from the analysis of that specific bone. Consequently, the final number of paired specimens, the female–male ratio, and the landmark count showed slight bone-to-bone variation. The sample sizes reported in Table 1 were therefore used in all subsequent analyses.

### 2.2. Modelling

Three-dimensional modelling of the long bones was carried out in 3D Slicer (version 5.2.2), starting from the DICOM datasets obtained by CT [21]. For each animal, the limb segments were loaded into Slicer and the target long bones were segmented separately for the left and the right side (humerus, antebrachium, femur, or crus) using thresholding followed by manual refinement. After clean segmentations were obtained, surface models were generated and exported as polygonal meshes (PLY format).

**Figure 1:**
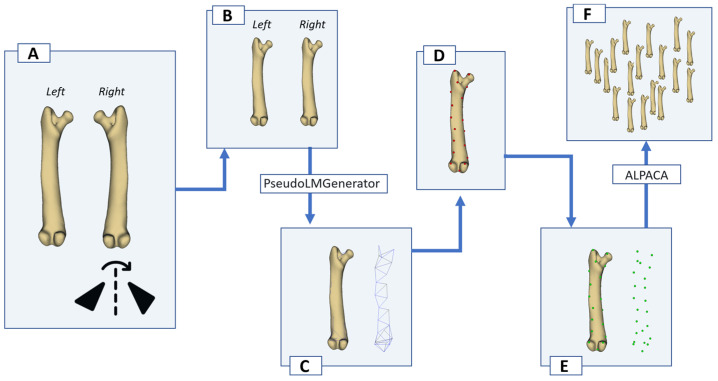
Workflow for generating and propagating pseudo-landmarks on guinea pig long bones. (**A**) Left and right bones are segmented separately; the right bone is mirrored (reflected) across the sagittal plane to match the left-sided orientation for comparison and automated landmarking. (**B**) Left and right models aligned in the same coordinate system. (**C**) A surface template is created on the reference model in 3D Slicer using the PseudoLMGenerator module (tolerance set to 10%). (**D**) Dense pseudo-landmarks are placed on the reference bone. (**E**) The reference model with its landmark set is used as the source for automated landmark transfer. (**F**) Using the SlicerMorph ALPACA module, the same landmark configuration is transferred to all remaining specimens of that bone.

For automated landmarking and for the asymmetry analyses, the right-side meshes were reflected (mirrored) across the sagittal plane so that their anatomical features were expressed in the same left-sided orientation as the reference meshes (Figure 1A). Importantly, the ‘mirrored right’ configuration represents the original right bone after reflection, not a synthetic copy generated from the left bone. After reflection, all meshes were brought into a common anatomical pose by aligning the long axis with the global proximal–distal axis, enforcing a consistent medio–lateral direction, and removing obvious rotations (Figure 1B). This standardization facilitates direct comparison of corresponding surface regions and improves the reliability of automated landmark transfer and subsequent Procrustes-based left–right comparisons.

### 2.3. Landmarking

For each long bone, an initial template of surface points was generated in 3D Slicer using the PseudoLMGenerator module of SlicerMorph (Figure 1C) [22]. This tool was used to create a dense set of pseudo-landmarks on the external surface of the reference model without assuming a priori anatomical (biological) homology, i.e., landmarks corresponded to “geometric” rather than “type I” landmarks (Figure 1D). The sampling tolerance was set to 10% for each bone, so that the point cloud would provide sufficiently even coverage of the humerus, antebrachium, femur and crus despite their different lengths and curvatures (Figure 1E). The template was created on a clean, well-segmented exemplar of that bone (one specimen per bone), and this specimen-specific pseudo-landmark configuration was taken as the source landmark set for subsequent transfers.

After the template had been created, it was propagated to all other specimens of the same bone using the ALPACA (Automated Landmarking through Point Cloud Alignment and Correspondence Analysis) module in SlicerMorph [23]. ALPACA performs a point-cloud-based alignment followed by deformable registration between the source mesh (the bone with the template landmarks) and each target mesh (the remaining .ply models), and then transfers the pseudo-landmarks to the target surface while preserving point-to-point correspondence. In this way, every individual humerus, antebrachium, femur and crus received the same number of landmarks in the same geometric order, which is a prerequisite for Procrustes-based shape, PCA, and asymmetry analyses (Figure 1F). The resulting landmark sets were exported in Slicer markup JSON format (.mrk.json) and constituted the dataset used in R. Specimens in which the pseudo-landmarks were visibly attracted to CT artefacts, irregular mesh regions, or mirrored surfaces were flagged and excluded from the analysis for that specific bone.

### 2.4. Statistical Analyses

All statistical analyses were carried out in R (version 4.4.2) using the package geomorph (v.4.0.9) [24,25]. All shape-related procedures were based on Generalized Procrustes Analysis (GPA), which was performed with the gpagen function of the geomorph package [26,27]. Each long bone (humerus, antebrachium, femur, and crus) was analyzed separately, and GPA for testing sexual shape dimorphism was run only on the original left bones. This was done deliberately to avoid inflating within-individual variance that may arise from including mirrored right elements in the same alignment. After Procrustes superimposition of the left elements, sexual shape dimorphism was assessed by means of a permutation-based Procrustes analysis of variance using the procD.lm routine in geomorph, with the model specified as “shape coordinates = sex”. For each bone, 999 permutations of the residuals were used, and the following statistics were recorded: degrees of freedom, sums of squares, F-statistic, proportion of variance explained by sex (R^2^), and permutation *p*-value. This model specifically tests whether female and male guinea pigs differ in mean bone shape, independent of right–left asymmetry.

To determine whether the observed shape differences were purely geometric or partly size-driven (i.e., allometric), centroid size (Csize) values returned by GPA were analyzed separately [10]. For each bone, centroid sizes of females and males were grouped, descriptive statistics (sample size, mean Csize, standard deviation) were computed, and sex differences in size were evaluated using a Welch two-sample *t*-test (*t*-test (Csize ~ sex)). This univariate step made it possible to distinguish shape differences accompanied by a systematic sex difference in overall bone size from those that reflect shape variation alone.

The structure of shape variation and the spatial arrangement of individuals were further explored by principal component analysis (PCA) of the GPA-aligned coordinates, again using only left elements. Multivariate PCA was performed with the corresponding function in geomorph, the percentage of variance explained by the first two components was extracted from the PCA summary, and scores were visualized with ggplot2. In these scatterplots, females and males were displayed with separate convex hulls (lightly shaded), so that the degree of overlap between the two sex groups could be inspected visually. Axis labels were kept as “PC1” and “PC2”, while the percentage of variance explained by each component was reported in the figure titles.

The asymmetry component of the study followed a separate but parallel workflow. For this part, left and mirrored right configurations of the same bone were first combined into a single three-dimensional array and jointly aligned by GPA, ensuring that the two sides of each individual were superimposed in the same Procrustes space. Here, ‘mirrored right’ refers to the reflected version of the independently segmented right element, used only to standardize orientation for left–right comparison. On these aligned coordinates, a multifactor Procrustes ANOVA was fitted with the model “shape coordinates = sex × side + individual”, again using 999 permutations. In this model, the factor side tests for directional asymmetry (systematic left–right bias), the factor sex tests for overall sexual shape differences, the interaction sex × side tests whether asymmetry itself differs between females and males (sex-specific asymmetry), and the factor individual accounts for the paired nature of the data (two sides measured for the same specimen). In parallel, individual fluctuating asymmetry (FA) was quantified by computing, for each specimen, the difference between left and mirrored right configurations, subtracting the grand mean left–right difference (directional component), and taking the Euclidean norm of the residual. These FA values were then assembled into a table and compared between sexes by Welch’s *t*-test when appropriate. Throughout the pipeline, data wrangling and file matching were handled with dplyr, tibble, and purrr, while the construction of landmark arrays of the form k × 3 × n was performed with abind.

## 3. Results

### 3.1. Sex-Related Differences in Shape and Centroid Size of Bones

In the left humerus, sexual shape dimorphism was the weakest among the four elements (R^2^ₛₑₓ = 0.048, *p* = 0.163), and the centroid-size comparison likewise suggested only a modest tendency for males to be larger; in other words, humeral shape differences between female and male guinea pigs were small and only partly size-related. In the left antebrachium (forearm), the sex effect on shape was significant (R^2^ₛₑₓ = 0.063, *p* = 0.046), and this signal was consistent with a slight male-biased increase in centroid size, indicating that part of the shape dimorphism in the forelimb was accompanied by a size component. The clearest size difference was observed in the left femur, where males had a distinctly greater centroid size than females (female: 67.5 ± 2.9; male: 70.8 ± 2.3) and this difference was statistically significant (Welch t = −3.24, *p* = 0.0041). Finally, in the left crus, the shape effect of sex was also significant (R^2^ₛₑₓ = 0.075, *p* = 0.016), and the pattern was compatible with males having slightly larger tibia–fibula complexes than females, although the magnitude of the size difference was smaller than in the femur.

### 3.2. Principal Component Analysis of Bones

PCA performed on the GPA aligned left elements showed that the first two components explained between 25 and 33% of the total shape variance, depending on the bone. The highest cumulative variance on PC1–PC2 was observed in the crus (32.7%) and antebrachium (31.8%), while the humerus showed an intermediate value (28.2%) and the femur the lowest (25.6%).

**Figure 2:**
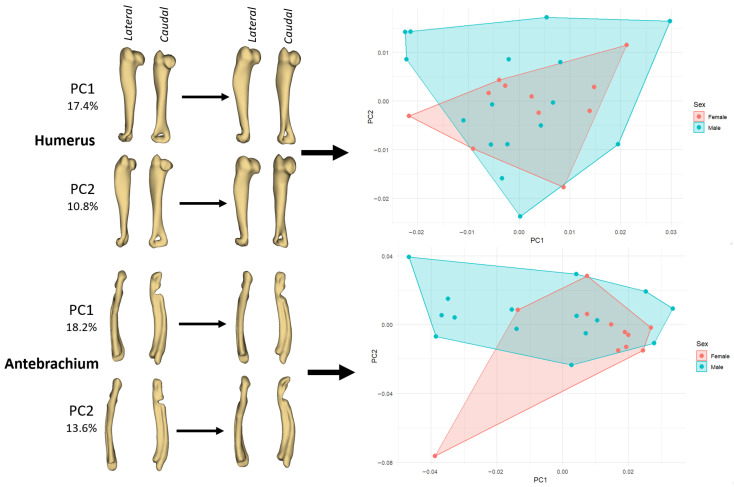
PCA of left long bones showing shape changes associated with the first two principal components and the corresponding ordination of female and male guinea pigs. **Left panels** illustrate deformation along PC1 and PC2 for the humerus (PC1 = 17.4%, PC2 = 10.8%) and antebrachium (PC1 = 18.2%, PC2 = 13.6%) in lateral and caudal views. **Right panels** show PC1–PC2 scatterplots with convex hulls drawn separately for females (pink) and males (cyan).

In the humerus, variation along PC1 (17.4%) primarily describes a change from a slenderer, slightly cranio-caudally compressed shaft with a relatively modest proximal epiphysis (negative PC1) to a more robust bone with a fuller deltoid/greater tubercle region and a broader distal end (positive PC1) (Figure 2). In PC2 (10.8%), the main trend is a reorientation of the distal extremity and a subtle torsion of the shaft, producing a shift in the caudal contour of the diaphysis. The PCA plot shows that males occupy a somewhat wider region of morphospace than females, but the two convex hulls overlap broadly, in line with the Procrustes ANOVA indicating only low-to-moderate sexual shape dimorphism.

In the antebrachium, PC1 (18.2%) captures the degree of longitudinal curvature and mediolateral bowing of the radius–ulna segment: specimens at the negative end exhibit a straighter, more gracile forearm, whereas those at the positive end show a more curved shaft and a relatively bulkier proximal part, suggesting functional or positional differences in the distal forelimb (Figure 2). PC2 (13.6%) mainly affects the distal segment, altering the relative width and orientation of the carpal/articular region and slightly changing the cranio-caudal profile of the ulna. As in the humerus, female and male antebrachia form overlapping clusters, but males tend to extend the morphospace towards the positive PC1/positive PC2 quadrant, which is consistent with males having slightly more robust or more curved forearms.

**Figure 3:**
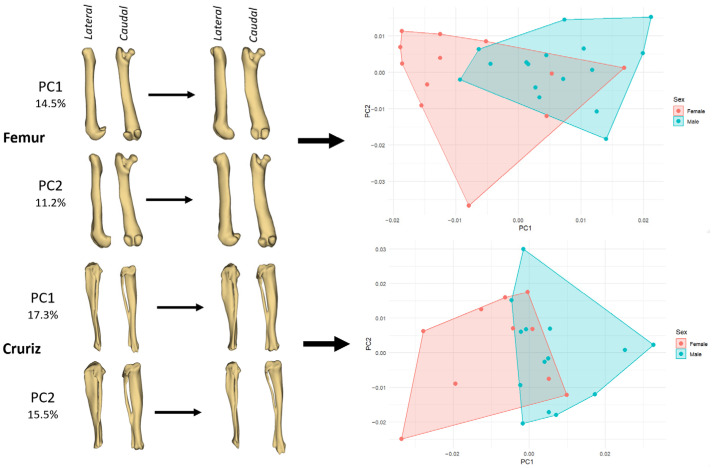
PCA of left hindlimb bones showing shape changes along the first two components and the distribution of female and male guinea pigs in morphospace. **Left panels** illustrate deformation patterns for the femur (PC1 = 14.5%, PC2 = 11.2%) and the crus (crus; PC1 = 17.3%, PC2 = 15.5%) in lateral and caudal views. **Right panels** show the corresponding PC1-PC2 scatterplots with convex hulls drawn separately for females (pink) and males (cyan).

In the femur, variation along PC1 (14.5%) mainly reflects a transition from a relatively slender diaphysis with a slightly narrower distal epiphysis (negative PC1) to a more robust shaft and a mediolaterally broader distal extremity (positive PC1) (Figure 3). This axis also captures minor changes in femoral curvature, with positive PC1 femora appearing slightly straighter in lateral view. PC2 (11.2%) modifies chiefly the proximal part of the bone, altering the prominence and orientation of the femoral head and greater trochanter, and inducing a subtle change in the cranio-caudal profile of the distal condyles. The PCA plot indicates that males span a somewhat wider portion of morphospace, particularly towards positive PC1 values, which is consistent with the centroid-size results showing larger femora in males; nonetheless, the two sex-specific hulls still overlap broadly.

In the crus (tibia–fibula segment), PC1 (17.3%) describes a change from a straighter, more gracile crus (negative scores) to a bone with a more pronounced longitudinal curvature and slightly thickened proximal third (positive scores) (Figure 3). PC2 (15.5%) affects mostly the distal segment, modifying the width and orientation of the distal articular region and producing a small shift in the caudal outline of the tibia. In the ordination plot, females tend to cluster in the negative PC1/near-zero PC2 region, whereas males extend the cloud towards positive PC1 and positive PC2, indicating that male guinea pigs more often exhibit the more curved and slightly bulkier crus shape. As with the forelimb bones, however, the overlap between the two sexes is substantial, underscoring that sexual shape dimorphism in these long bones is present but not strongly discrete.

### 3.3. Right–Left Asymmetry (Directional and Fluctuating) in Guinea Pig Long Bones

As a result of the left–right (mirrored) asymmetry analysis of the humerus, the Procrustes ANOVA revealed a significant main effect of sex (R^2^ = 0.034, *p* = 0.002), suggesting that male and female guinea pigs differed in overall humeral shape despite the use of a denser landmark configuration (28 landmarks) (Table 2). However, the effect of side itself was not significant (*p* = 0.227), and the sex × side interaction was likewise not significant (*p* = 0.810). These findings indicate that right–left asymmetry in the humerus is weak and not sex-dependent, and that most of the variation in this bone is better explained by individual differences than by directional or sex-specific asymmetry.

As a result of the left–right (mirrored) asymmetry analysis of the antebrachium, individual shape differences were present but the overall asymmetry pattern was weak (Table 3). The Procrustes ANOVA showed that the main effect of sex was significant (R^2^ = 0.040, *p* = 0.002), indicating that female and male guinea pigs differed in overall antebrachium shape even though 21 landmarks were used. However, the effect of side was not significant (*p* = 0.802), and the sex × side interaction was also non-significant (*p* = 0.834), which means that right–left asymmetry in this bone did not systematically vary between sexes. Taken together, these results suggest that most of the observed variation in the antebrachium arises from individual-level differences rather than directional asymmetry.

As a result of the left–right (mirrored) asymmetry analysis of the femur, a significant main effect of sex was detected (R^2^ = 0.044, *p* = 0.001), showing that male and female guinea pigs differed in their overall femoral shape when 23 landmarks were considered (Table 4). In contrast, the side effect did not reach significance (*p* = 0.184), and the sex × side interaction was clearly non-significant (*p* = 0.583). This indicates that the magnitude and direction of right–left asymmetry in the femur were generally low and did not differ between sexes. Accordingly, the pattern observed for the femur is consistent with low directional asymmetry and a predominance of individual-level shape variation.

As a result of the left–right (mirrored) asymmetry analysis of the crus bone, individual fluctuating asymmetry (FA) values were found to range between 0.03 and 0.05 (Table 5). Although the mean FA in female guinea pigs (0.0449 ± 0.0086) was higher than in males (0.0388 ± 0.0066), this difference was slightly above the statistical threshold according to the Welch *t*-test (t = 1.88, df = 16.3, *p* = 0.079). Therefore, it can be suggested that there is a tendency toward greater asymmetry in females, but this cannot be demonstrated conclusively with the current sample size. The Procrustes ANOVA results also supported these findings: the main effect of sex was significant in the model (R^2^ = 0.048, *p* = 0.001), indicating an overall shape difference between female and male guinea pigs in the crus bone; however, the effect of side was not significant (*p* = 0.873), and the sex × side interaction was only weak (*p* = 0.108).

In all Procrustes ANOVA tables, sex denotes the biological sex of the guinea pigs and tests for overall sexual shape dimorphism; side represents the mirrored left–right factor and tests whether there is a consistent directional asymmetry in the bone; ind (individual) accounts for repeated observations from the same specimen and therefore captures the main source of among-individual shape variation; and sex:side tests whether the magnitude or pattern of right–left asymmetry differs between females and males (i.e., sex-specific asymmetry). Residuals correspond to the portion of shape variation that is not explained by these factors and can be attributed to measurement error or other unmodelled sources (Table 2, Table 3, Table 4 and Table 5).

**Figure 4:**
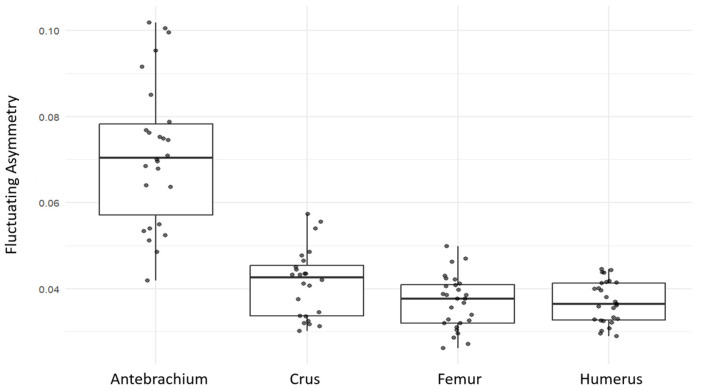
Distribution of fluctuating asymmetry values in four long bones (antebrachium, crus, femur, humerus) of guinea pigs. Boxplots show the median and interquartile range; points represent individual specimens.

When fluctuating asymmetry values were compared across the four long bones, the antebrachium showed the highest level of asymmetry (mean FA = 0.0716 with 21 landmarks), clearly higher than the other bones (Figure 4). The crus ranked second (mean FA = 0.0414 with 23 landmarks), while the humerus and femur exhibited the lowest and very similar FA values (mean FA = 0.0369 with 28 and 23 landmarks, respectively).

## 4. Discussion

The present study demonstrates that 3D geometric morphometric methods can be applied reliably to guinea pig long bones when CT-based models, standardized mirroring, and a pseudo-landmark/ALPACA workflow are combined. By generating a single template of pseudo-landmarks in Slicer and transferring it to all specimens of the same bone, we were able to enforce geometric correspondence across individuals even in the absence of clear type-I anatomical points. This is important for small mammal bones, where epiphyseal contours are smooth and muscular insertions are not always sharply delimited on CT-derived meshes. The fact that only a few specimens had to be removed due to reconstruction artefacts or erroneous landmark attraction indicates that the pipeline is robust and can be reproduced for other appendicular elements or closely related species.

A consistent outcome across all four bones was that sex explained a small but non-negligible portion of shape variation. Procrustes ANOVA on left elements showed R^2^ values in the range of ~5–7% for the antebrachium, femur and crus, while the humerus showed the weakest signal. This pattern fits well with the centroid-size results: where males were clearly larger (most obviously in the femur), they also tended to occupy the “more robust” region of morphospace in PCA. Conversely, in the humerus, where size dimorphism was modest, shape dimorphism was also modest. In other words, part of the sexual shape difference appears to be allometric, but not entirely so, PCA plots show that males often extend the cloud rather than forming a separate cluster, suggesting that males express the extremes of a shape continuum already present in females.

The asymmetry results were clearly not homogeneous across the limb, and the forearm (antebrachium) was the element in which asymmetry was most expressed. Taken together, the four bones suggest a simple gradient: bones that are farther from the trunk and functionally more versatile tended to show higher fluctuating asymmetry, whereas more proximal elements were comparatively symmetrical. Because the antebrachium is both distal and part of the forelimb, which in guinea pigs is used not only for locomotion, like the hind limb, but also for grasping, food acquisition and postural support, its greater asymmetry is biologically plausible. In other words, the combination of distance from the body axis and multifunctional use seems to increase small, random left–right departures. On this basis, it can be hypothesized that forelimb bones, especially those positioned distal to the elbow, are more prone to accumulate developmental or use-related asymmetry than the femur or the proximal hind limb, and that asymmetry may increase gradually as we move away from the body centre.

Given that the forelimb bears a greater proportion of body weight and makes earlier contact with the ground, it is reasonable to assume that bones in the anterior limb experience more variable loading, which could contribute to small right–left discrepancies over time. However, our data also show that asymmetry cannot be explained by “greater loading in front” alone. The highest FA was observed in the antebrachium, and in this bone, the increase can plausibly be linked not only to weight bearing but also to its broader functional repertoire (food handling, positioning, postural support). This explanation works well for the zeugopodial elements, but it does not fit the stylopodium to the same extent: FA values for the femur and humerus were very similar, and we did not detect an anterior excess of asymmetry there.

Reeves’s study on cotton-top tamarins showed that true directional asymmetry in long bones is quite limited, whereas fluctuating asymmetry is widespread across most measurements, and that this FA follows a hierarchical distribution within the bone, increasing toward the diaphysis and articular regions [12]. Our guinea pig data corroborate this general picture: directional asymmetry was weak, but FA was clearly present. What distinguishes our study is that we were able to show that FA does not only vary within a single bone but also increases gradually between long bones themselves. In other words, the observation by Reeves et al. that “regions subjected to greater functional or torsional loading tend to be more asymmetric” appears in our material at a broader anatomical scale as “bones that are farther from the trunk and used more multifunctionally tend to be more asymmetric” [12]. Their pattern emerged within the bone, toward the shaft, whereas ours emerged across the limb, toward the distal segments. Taken together, this suggests that long-bone asymmetry in small mammals is not merely measurement noise but can follow consistent biomechanical/functional gradients at both micro (within-bone) and macro (between-bone) levels. Similarly, in humans, especially in sports that place repeated load on one side of the body (e.g., handball, tennis), marked right–left differences in trunk and limb muscle mass as well as isometric strength have been reported, supporting the view that asymmetry is sensitive to repetitive, unilateral loading [28,29].

In several historical and archeological human samples, high bilateral asymmetry in the humeral diaphysis has been shown to concentrate in the midshaft, where bending and torsional loads are greatest, and to decrease toward both the proximal and distal ends, indicating that mechanical loading is a primary determinant of asymmetry [30,31]. The fact that, in our guinea pig dataset, we observed the gradient not within a single bone but between different long bones does not contradict this interpretation; rather, the finding that segments exposed to greater loading and functional diversification (particularly the antebrachium) are more asymmetric suggests that the mechanically sensitive asymmetry model described for the human humerus can be extended to the forelimb of small mammals. The key difference is that the human study captures large asymmetries produced by strong, unilateral behaviours (such as atlatl throwing), whereas our study reveals finer-scale asymmetries arising from more routine differences in limb use.

In this study, PCA of the left long bones from both the fore- and hindlimb showed that the first two components explained a relatively limited yet interpretable portion of shape variation: for the humerus, PC1 explained 17.4% and PC2 10.8%; for the antebrachium, PC1 explained 18.2% and PC2 13.6%; for the femur, PC1 explained 14.5% and PC2 11.2%; and for the crus, PC1 explained 17.3% and PC2 15.5% of the variation. Intraspecific datasets that are built from the same landmark template are expected to show this level of variance on the first axes, and this pattern indicates that the sample is morphologically homogeneous and that most differences are of a fine scale [11,32]. This, in turn, supports the reliability of the data used for the asymmetry analyses, because in asymmetry studies, part of the observed right–left differences may arise from operator error in landmark placement, and specimens are therefore often landmarked twice to separate measurement error from true morphological asymmetry [7,9]. In our case, however, the use of computer-assisted/automatic landmark transfer and the fact that Procrustes distances were very similar across specimens suggest that the right–left differences we detected largely reflect true shape variation, with measurement error playing a minor role. Consequently, the limited but consistent variance structure observed in the PCA can be regarded as additional evidence for the robustness of our asymmetry results.

Some limitations of this study should be considered when interpreting the findings. First, all analyses were carried out in a single species and only in adult individuals; however, during the growth period (juvenile–subadult stages) both long-bone shape and loading patterns are more dynamic, and developmental instability may be more pronounced. A future comparison of different age classes using the same protocol would help clarify when asymmetry emerges and whether it stabilizes in adulthood. Second, asymmetry was interpreted here solely on the basis of morphometric data; if functional information such as gait analysis, ground reaction forces, or segment-specific load distribution had been available, the distal increase in asymmetry could have been linked more directly to actual movement patterns. For this reason, future studies that collect 3D morphometric and functional/biomechanical data on the same animals will be better positioned to explain why the antebrachium, in particular, shows higher FA values.

## 5. Conclusions

In conclusion, the findings indicate that asymmetry in guinea pig limb bones does not result from a single developmental accident but emerges gradually along the limb, in close association with bone position and functional demand. Distal, multifunctional forelimb elements, especially the antebrachium, showed higher fluctuating asymmetry than proximal stylopodial bones, supporting the view that greater mobility and more diverse loading patterns promote small right–left departures. At the same time, sex accounted for a modest but consistent part of shape variation, and in some bones, this was accompanied by size differences, suggesting that sexual dimorphism and asymmetry act as partly independent sources of morphological variation. Taken together, these results highlight distal forelimb bones as key targets for future asymmetry work in small mammals and confirm that 3D geometric morphometrics is sensitive enough to detect these subtle patterns.

## Figures and Tables

**Table 1:** Sample size, sex composition, and landmark configuration for guinea pig long bones included in the asymmetry analyses.

Bone	Paired Specimens	Female (Paired)	Male (Paired)	Landmarks Used
Humerus	27	11	16	28
Antebrachium	26	11	15	21
Femur	28	12	16	23
Crus	24	10	14	23

**Table 2:** Procrustes ANOVA for right–left asymmetry in the Humerus (28 landmarks).

Effect	SS	MS	R^2^	F	*p*	Interpretation
sex	0.00181	0.00181	0.0336	2.14	0.002	sex-related shape difference
side	0.00098	0.00098	0.0181	1.16	0.227	side not significant
individual	0.02348	0.00130	0.4351	1.54	0.001	strong among-individual variation
sex:side	0.00064	0.00064	0.0119	0.76	0.810	no sex-dependent asymmetry
Residuals	0.02705	0.00085	0.5014	—	—	—

**Table 3:** Procrustes ANOVA for right–left asymmetry in the Antebrachium (21 landmarks).

Effect	SS	MS	R^2^	F	*p*	Interpretation
sex	0.00697	0.00697	0.0399	2.31	0.002	shape differs between sexes
side	0.00227	0.00227	0.0130	0.76	0.802	no main side effect
ind	0.07278	0.00404	0.4173	1.34	0.001	strong among-individual variation
sex:side	0.00208	0.00208	0.0120	0.69	0.834	sex does not change asymmetry pattern
Residuals	0.09031	0.00301	0.5178	—	—	—

**Table 4:** Procrustes ANOVA for right–left asymmetry in the Femur (23 landmarks).

Effect	SS	MS	R^2^	F	*p*	Interpretation
sex	0.00245	0.00245	0.0441	2.88	0.001	sex-related shape difference
side	0.00103	0.00103	0.0184	1.20	0.184	side not significant
ind	0.02253	0.00125	0.4043	1.47	0.001	strong among-individual variation
sex:side	0.00077	0.00077	0.0138	0.90	0.583	no sex-dependent asymmetry
Residuals	0.02894	0.00085	0.5194	—	—	—

**Table 5:** Procrustes ANOVA for right–left asymmetry in the Crus (23 landmarks).

Effect	SS	MS	R^2^	F	*p*	Interpretation
sex	0.002697	0.0026975	0.04795	2.7226	0.001	Significant main effect of sex
side	0.000694	0.0006944	0.01234	0.7008	0.873	No main effect of side (no strong DA)
ind	0.024695	0.0014526	0.43895	1.4661	0.001	Strong among-individual variation
sex:side	0.001421	0.0014207	0.02525	1.4339	0.108	Sex-specific asymmetry only weak/marginal
Residuals	0.026751	0.0009908	0.47551	—	—	—

## Data Availability

The data presented in this study are available upon request from the corresponding author (O.G.).
